# Dental Therapy of Patients Prior to Endoprostheses: A Retrospective, Telephone-Based Cohort Study

**DOI:** 10.3390/dj11080198

**Published:** 2023-08-18

**Authors:** Gerhard Schmalz, Julian Wirtz, Rainer Haak, Fabian Fenske, Andreas Roth, Dirk Ziebolz

**Affiliations:** 1 Department of Cariology, Endodontology and Periodontology, University of Leipzig, 04103 Leipzig, Germany; julian.wirtz@gmx.de (J.W.); rainer.haak@medizin.uni-leipzig.de (R.H.); dirk.ziebolz@medizin.uni-leipzig.de (D.Z.); 2 Department of Maxillofacial Surgery, University of Leipzig, 04103 Leipzig, Germany; fabian.fenske@medizin.uni-leipzig.de; 3 Department of Orthopaedics, Trauma and Plastic Surgery, University Hospital Leipzig, 04103 Leipzig, Germany; andreas.roth@medizin.uni-leipzig.de

**Keywords:** oral health, endoprosthesis, oral health behavior

## Abstract

The aim of this study was to assess, whether patients prior to endoprosthesis (EP) visit their dentist for need-oriented therapy and whether this would be associated with the occurrence of complications. Based on a cohort of patients, which was orally investigated prior to EP surgery between 04/2020 and 12/2021, a telephone interview was performed at least six months after EP implantation. Patients were classified into either low-risk (LR), moderate-risk (MR), or high-risk (HR) groups. Participants were interviewed based on a structured questionnaire regarding dental visits, dental therapy, and potential complications during the observational period. Out of the 311 patients from the baseline cohort, 96 patients after EP implantation could be included (participation rate of 31%). Nineteen patients were in LR (20%), 41 in MR (43%), and 36 in the HR group (37%). Overall, 79% (*n* = 76) of the patients followed the recommendation to visit their dentist; 94% of patients within the HR group visited the dentist (*p* = 0.02). Dental treatment procedures included tooth cleaning (57%), periodontal treatment (31%), restorative therapy/filling (28%), and tooth extraction (28%). In 64% of the HR patients (*n* = 23), the potential oral foci with a risk of EP infection were eliminated by their general dentist. Fourteen different complications occurred within the observation period, without any group effect (*p* > 0.05). In conclusion, most patients prior to EP visit their general dentist following referral, especially if they have a potential oral focus. The effect of dental clearance on infectious complications of EP remains unclear, whereby further clinical studies are needed.

## 1. Introduction

The reduction of complications of endoprostheses (EP) of the hip and the knee due to infections is of high clinical importance, given their enormous morbidity and economic burden. In this context, the oral cavity has a potential role in the development and thus in the prevention of periprosthetic joint infections [1]. Accordingly, the role of dental care, oral diseases, especially potential oral foci of EP infection. is repeatedly discussed in the literature [2,3,4,5].

The potential risk of invasive dental procedures for the development of EP infections is discussed. Thereby, the recent literature indicated that there is no increased risk for EP infections after invasive dental procedures [6]. Moreover, antibiotic prophylaxis for dental treatment after EP implantation has been reported to bring no benefit on the reduction of periprosthetic infections [5,6,7]. Consequently, the only plausible approach to reduce EP infections from the perspective of dentistry appears to be a preoperative dental assessment and respective need-oriented therapy. Although the evidence is limited, a dental examination and respective maintenance seems recommendable prior to EP surgery [3]. However, there is inconsistent knowledge regarding appropriate concepts for dental referral, screening, and therapy before EP implantation. A previous study introduced a comprehensive dental referral concept in a university setting, which applied a risk classification system [8]. Nevertheless, there are several difficulties within such a concept. Patients prior to EP focus on their forthcoming surgery and often show a certain neglect of oral health [9]. Different issues could be of relevance in this context. Oral disease might develop in the period of waiting for assessment for the EP or could also reflect premorbidity within the population likely to require prostheses, such as gradual loss of independence and increased frailty amongst those more likely to need endoprosthetic replacement. This is reflected in the high prevalence of oral diseases, like periodontitis, tooth decay or other oral inflammations in those patients [10,11]. On the other hand, patients prior to EP often show a reduced oral health behavior, insufficient information, and orthopedic centers rarely cooperate with respective dentists, what appears a general problem in the interprofessional cooperation between dentists and other medical professionals [9,12,13]. In this respect, different barriers are conceivable, including limited timeframes, financial issues, or co-morbidities, what might increase the complexity in this cohort. Taken together, patients prior to EP surgery often show a high prevalence of oral diseases, reduced oral health behavior, and thus a need for dental referral, whereby current concepts are not validated appropriately. Accordingly, there is a research gap regarding the utilization of dental therapy of patients prior to EP, especially in context of potential complications after surgery (e.g., periprosthetic infections).

Therefore, this current study focused on a cohort, which has been referred to their general dentist prior to EP surgery, depending on a risk classification [8]. The research objective of the current study was to evaluate the dental therapy prior to EP surgery after a risk-oriented dental referral. Thereby, it should also be evaluated whether the performed dental procedures would be associated with the respective risk class as well as with the occurrence of complications (e.g., EP infections). For this, telephone-based, structured interviews were performed with patients, who had received a dental examination and referral to their general dentist prior to EP surgery. It was hypothesized that most patients had visited the dentist, who had performed the need-oriented therapy, accordingly. A side hypothesis was that the risk class and the absence of dental therapy would be associated with postoperative complications.

## 2. Materials and Methods

### 2.1. Study Design

This retrospective, interview-based cohort study followed a previous cohort investigation of this working group based on 311 patients before EP implantation [8]. For this current study, those patients should be evaluated in follow-up after their EP surgery. The study protocol was checked and approved by the ethics committee of the Leipzig University (No: 116/20-ek). All patients were informed orally and in writing and gave their written informed consent; moreover, the study protocol followed the Declaration of Helsinki. 

### 2.2. Patients

Patients, who had received orthopedic surgery for hip or knee replacement at the Department of Orthopedics, Trauma and Plastic Surgery, University Hospital Leipzig between 04/2020 and 12/2021 were checked regarding their eligibility according to the following inclusion and exclusion criteria:

Inclusion criteria: first hip or knee EP replacement (no revision surgery) and participation in a previously performed dental consultation concept, including comprehensive oral examination and risk classification for oral disease-related EP infection at the Department of Cariology, Endodontology, and Periodontology, University Hospital Leipzig [8]. Furthermore, based on risk classification (high risk) and dental treatment need, a recommendation for the general dentist was formulated, including the required therapy. The EP surgery should be at least half a year ago. 

Exclusion criteria: 

(1) Equal criteria as for the previous study [8], as it was mandatory to participate in this previous examination. Specifically, for this current study. 

(2) Lack of initial consent to participate in this study part (contact by phone) and EP retreatment (revision), infection, or joint replacement (first or second step change).

No sample size calculation was performed previously. The included individuals in the previous cross-sectional study limited the potential number of patients for this current study. It was aimed to include as many patients from the original cohort in the follow-up as possible. 

### 2.3. Data Collection

Patient-related data were assessed from the patient’s records: 

(a) Age, gender, date, and form of EP implantation (knee or hip), complications in hospital setting until four months after EP implantation (e.g., infection, wound healing disorder (WHD), and loosening). 

(b) Dental and periodontal treatment need with specific clinical and radiographic dental or dento-alveolar findings (oral foci) as well as risk classification (low, moderate, and high).

### 2.4. Risk Classification

The risk classification was initially described in a previous study [8]. In brief, based on the dental, periodontal, and radiographical examination, the risk of patients to get a periprosthetic infection with oral cause was determined. Therefore, patients without oral foci, dental, and periodontal treatment need were classified as of low risk. Patients with dental and/or periodontal diseases, but without oral foci were classified as of moderate risk. Patients with any kind of oral focus (e.g., severe periodontitis with suppuration, teeth with apical radiolucency, and caries penetrans) were categorized into the high-risk group. The risk classification was performed once within the initial dental consultation, as described previously [8]. 

### 2.5. Interview Manual

Between 11/2021 and 06/2022, included patients were called and a telephone interview based on a structured manual was conducted. A structured interview manual, including the following items was composed: 

(I) Dental contact before and after EP implantation based on the specific recommendation and information about performed dental treatment by general dentist. The dental information included dental treatment, preventive measures (only professional tooth cleaning), periodontal treatment (scaling and root planning), restorative treatment, and tooth extraction. 

(II) Complications after EP implantation (based on patients’ records and statement).

(III) Patient information: individual opinion about dental examination before EP implantation and oral health situation in context with EP prophylaxis as well as dentists’ knowledge and handling. All of the obtained dental information were self-reported by the patients. 

The manual was composed by dentists and orthopedists as well and has been checked within a short pre-test, whereby the interview questions were asked to five selected patients. After this, the questions were modified, if necessary, to ensure appropriate understandability. All interviews were performed by one experienced orthopedist, which has been trained prior to the study in providing the telephone interviews based on the standardized interview manual.

### 2.6. Study Flow

As described previously, patients before EP implantation from the Department of Orthopedics, Trauma, and Plastic Surgery, University Hospital Leipzig were referred for a dental examination in the Department of Cariology, Endodontology, and Periodontology, University Hospital Leipzig. The dental examination included detection of dental and periodontal treatment need as well as dento-alveolar or oral foci based on intra-oral and radiographic findings. Based on these findings, patients were classified regarding the upper mentioned classification system: low risk (LR group), moderate risk (MR group), or high risk (HR group). All patients, especially of the HR group, received a doctor’s letter to their general dentist with information on oral and radiographic findings, and for HR group, a recommendation for dental treatment prior to EP surgery was included. 

At a minimum of six to eight months after EP implantation, patients of all three groups were called and interviewed based on the manual. Therefore, patients were called once for the respective interview; if patients were not available, two further attempts were made to contact the patients. 

### 2.7. Statistical Analysis

The statistical analysis was performed with SPSS for Windows (Version 24.0, SPSS Inc., Armonk, New York, USA). Normal distribution of metric variables was checked with Kolmogorov–Smirnov Test. 

For comparison of more than two non-normally distributed independent samples, Kruskal–Wallis test was used, for more than two normally distributed independent samples, the single-factor ANOVA was applied. Categorized or nominal data were analyzed with Chi-Quadrat Test. The significance level was set at *p* < 0.05.

## 3. Results

### 3.1. Patients

**Figure 1:**
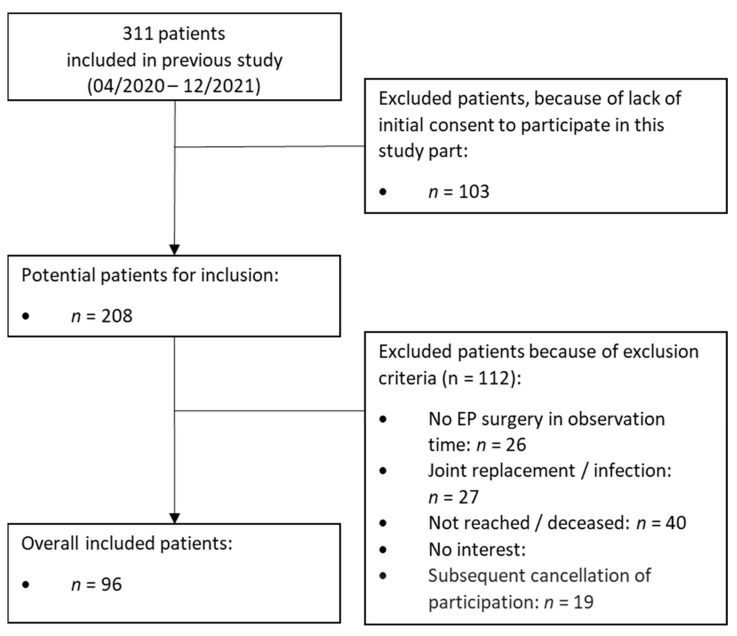
Overview of included patients under consideration of inclusion and exclusion criteria.

Out of the 311 patients, which had been examined in the baseline cohort [8], 96 patients after EP implantation could be included in this current study (participation rate of 31%). Reasons for non-participation in this study were lack of initial consent to participate (*n* = 103) and existing of one or more exclusion criteria and/or patients not being available via phone call (*n* = 112; Figure 1). The mean age of the included patients was 67.6 ± 10.1 years and 53% of the participants had male gender; 66% of the patients received a hip EP (Table 1). Based on the risk classification, 19 patients were assigned in the LR (20%), 41 in the MR (43%), and 36 in the HR group (37%; Table 1).

### 3.2. Dental, Periodontal, and Oral Surgical Treatment Need 

**Figure 2:**
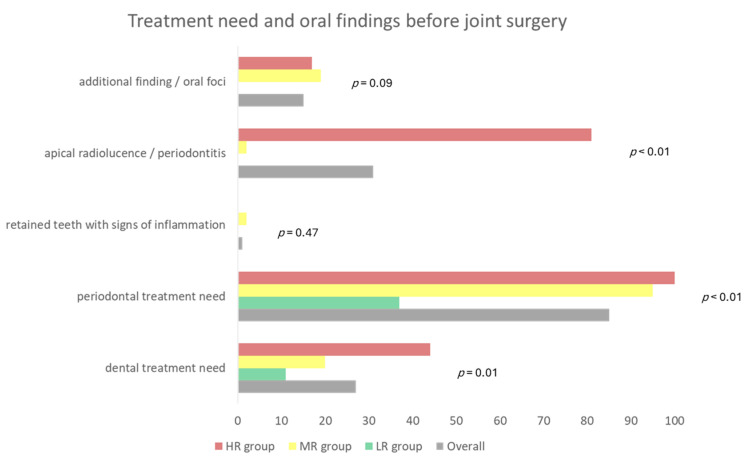
Treatment need (dental and periodontal) as well as selected oral findings prior to joint surgery in the included cohort. Values are given as percentage. (LR: low risk, MR: moderate risk, HR: high risk).

The oral findings of the initial dental examination before joint surgery (EP implantation) are shown in Figure 2. In the overall cohort, most oral or radiological findings were periodontal treatment need (85%), apical radiolucency (31%), and dental treatment need (27%); in the HR group, the highest amount of patients had periodontal (100%) or dental treatment need (44%) as well as apical radiolucency in a kind of apical periodontitis were found (81%). 

### 3.3. Dental, Periodontal and Oral Surgical Treatment by Family Dentist

**Figure 3:**
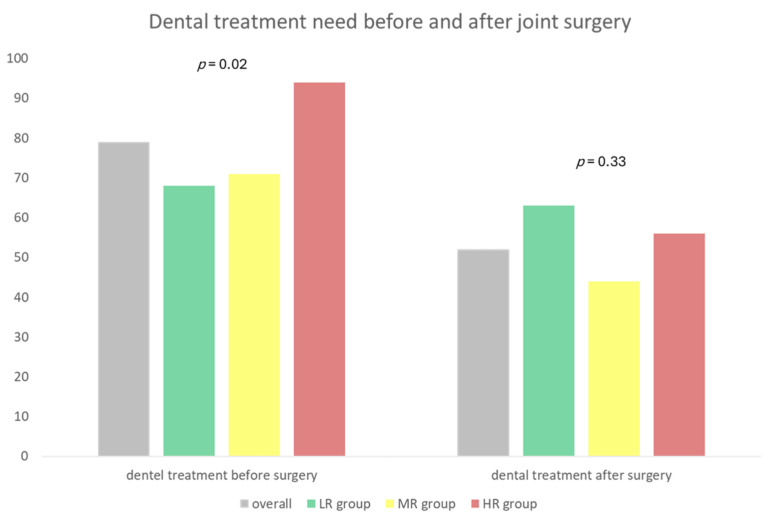
Dental treatment needs before and after joint surgery. Values are given as percentages. (LR: low risk, MR: moderate risk, HR: high risk).

**Figure 4:**
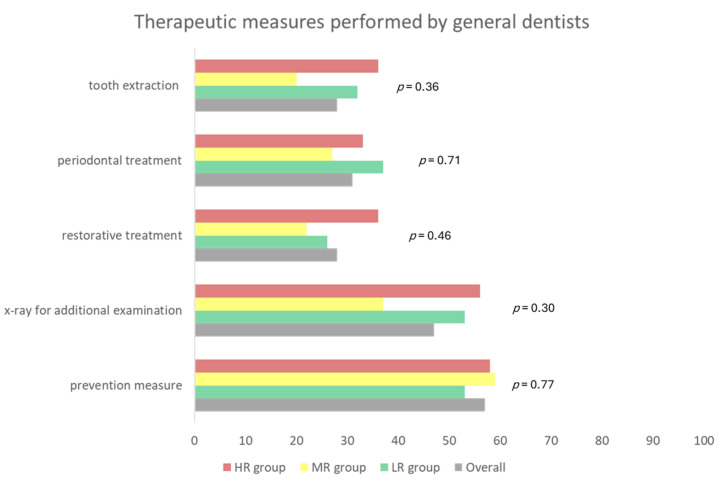
Therapeutic measures, which were performed by the respective general dentist prior to joint surgery in the included cohort. Values are given as percentages. (LR: low risk, MR: moderate risk, HR: high risk).

Overall, 79% (*n* = 76) of the patients followed the recommendation to seek their general dentist advice before EP implantation; out of this sample, 94% of patients in the HR group visited the dentist (Figure 3; *p* = 0.02). The most common dental treatment procedures based on determined treatment need were tooth cleaning (57%), periodontal treatment (31%), and restorative therapy/filling (28%) as well as tooth extraction (28%); no significant group effect could be found (Figure 4; *p* > 0.05). Comparing the detected treatment need at baseline dental examination with the self-reported dental measures, in 64% of the HR patients (*n* = 23), the treatment need of oral foci with a risk of EP infection was eliminated by the general dentist. After EP implantation, 52% of the patients had further treatment by the general dentist, whereof 56% were HR patients (Figure 4).

### 3.4. EP Complication

Overall, 14 different complications occurred within the observation period. Those include four infections (all without oral foci), one loosening, and nine others (minor problems), without any group effect (Table 2; *p* > 0.05). One case of infection was detected in the HR group; this patient did not follow the recommendation to visit the general dentist but had also no dental cause of infection. In nine cases, a medication or surgical intervention was performed: antibiosis (*n* = 4), surgical (re-)treatment (*n* = 4), and EP change (*n* = 1). A group effect could not be found (*p* > 0.05).

### 3.5. Patient Perspective

72% (*n* = 69) of the interviewed patients stated that it would be important to visit the dentist prior to EP implantation (LR: *n* = 15, MR: *n* = 32, HR: *n* = 22; *p* = 0.36). 86% (*n* = 83) considered oral health to be important in the context of EP (LR: *n* = 15, MR: *n* = 36, HR: *n* = 32; *p* = 0.25). 81% (*n* = 78) of the patients stated that they want intensive care from the general dentist with EP patients, thereof 86% of the HR group.

## 4. Discussion

The majority of patients, who had been referred to their general dentist, had visited the dentist prior to EP surgery; this was especially given in the HR group (94%). Mainly, preventive and/or periodontal measures were performed, while 28% of patients underwent tooth extraction. In nearly two thirds of the HR patients, dental referral led to the clearance of the oral foci. The risk group was not associated with any kind of postoperative complications. 

Against the background of the potential role of the oral cavity as a source of bacterial dissemination, considering the risk of periprosthetic infections, a dental screening prior to EP implantation appears recommendable [1,3]. One current large-scaled study evaluated that dental history was related to complications and costs after EP surgery, supporting that preoperative dental examinations are useful [14]. Therefore, a dental medical history, physical examination, and radiographs should be evaluated [15]. Furthermore, increasing efforts, costs, and a high treatment need appear to be worth considering in this context [16]. Overall, preoperative dental screening and need-based therapy before EP implantation seem reasonable; accordingly, the basic approach, which has been applied in the previous study [8] and presents the basis of the current evaluation in this study, is supported by the available literature. 

Out of the current sample of 96 patients at least six months after EP implantation, 37% were in the HR group, which means that they had at least one potential oral focus of EP infection before EP surgery. This argues for a high dental treatment need in the cohort, which is also in line with the literature [10,11,16]. As a strategy to solve this need for dental clearance, patients were referred to their general dentist for need-based therapy. It reads quite positive, that nearly all of HR patients and the majority of the overall cohort followed the recommendation. Against the background of reduced dental behavior, insufficient communication between dentists and orthopedists as well as a subjectively lowly perceived importance of oral health issues by patients before EP [9], this result is promising. In a previous study on patients with severe heart diseases, a comparable amount of patients visited the dentist after structured referral [16]. However, the previous study revealed that only a minority of patients had received appropriate dental rehabilitation [17]. While the current study did not clinically investigate the patients during follow-up, patients were interviewed regarding their dental therapy. It was thereby conspicuous that more than one-third of HR patients did not report a sufficient dental clearance, i.e., the removal of the detected oral foci. This appears to be in line with the previous study on patients with heart diseases. 

In this respect, a couple of issues require consideration. First, dental and especially periodontal treatment needs are high in German individuals over 60 years of age (a similar age group as in the current study) [18]. This indicates deficits in dental care, irrespective of the need for an EP. Second, a recent meta-analysis reported on dental service utilization; it was reported that poorer overall dental health, as well as several sociodemographic factors, could negatively affect the utilization of dental services [19]. Thus, patients prior to EP surgery could also have a decreased utilization of dental services, because they are impaired in their general health, in oral health (see above), and potentially in their sociodemographic parameters (e.g., reduced social support). This is somewhat similar to the third issue, i.e., the perception of oral status by the patients themselves. Patients with severe general disease show a response shift regarding the perception of oral health issues. This has already been confirmed for the cohort of patients prior to EP [9]. Considering all of those facts, a dental referral alone appears inappropriate to solve the high dental treatment need prior to EP, which is supported by the current study’s findings. Overall, improved strategies to foster oral health in those patients might be needed. Different approaches could thereby be promising, including increased oral health and oral hygiene education in caregivers, as already shown for the elderly [20]. Similar to patients with cardiovascular diseases, health promotion activities could be a promising strategy too [21]. Additionally, novel and timely approaches, e.g., using mobile apps for oral health promotion could be another way to support patients [22].

Regardless of those patient-based issues, general dentists must be considered as an important factor. The interprofessional collaboration between dentists and physicians is of high relevance in this context and appears an important challenge [23]. However, there are often differences in knowledge on interdisciplinary topics between dentists and physicians [24]. Moreover, dentists and physicians often have different views on their responsibility and issues of collaboration [12,13]. Therefore, it remains unclear, whether the deficits in the current study can be explained either by the patients or by the dentist, or even by both. Considering the upper mentioned study on patients with heart diseases [17] and the fact that the vast majority of HR patients reported that they had visited the dentist, there appears to be an unknown but reasonable deficit among the respective dentists. This, however, remains speculative based on the current study. Nonetheless, it appears relevant to consider whether dental referrals would increase the likelihood of compliance with recommendations to get a dental clearance. The results of this current study show that the referral concept led to reduced but not fully eliminated treatment needs. Potentially, there is some significance to medical doctors advising patients of the importance of dental care and helping them understand this to increase patients’ willingness to undergo dental therapy prior to EP surgery. This issue would be reasonable for future research in the field.

A further issue, which requires discussion, is the potential effect of preoperative dental screening on the risk of (infectious) complications at EP. This current study did not show any association between risk groups. Recent literature states that there is still limited evidence on the potential benefit of a preoperative dental screening on EP infections, although it appears to be a reasonable approach [1,3]. The current study’s findings must be interpreted against the background that EP infections are rare events, as only 0.3−2% of patients are reported to get an EP infection, of which only 3–13% could be of oral origin [3,25]. Obviously, the sample size in the current study is too low to make meaningful conclusions on the potential role of risk classification in dental care. Taken together, picking up the hypotheses of this current study, the main hypothesis that most patients had visited the dentist, who had performed the need-oriented therapy, can be partly confirmed. The side hypothesis that the risk class and the absence of dental therapy would be associated with postoperative complications cannot be confirmed, which is limited by the low sample size.

Strengths and limitations: this interview-based study investigated an issue of clinical relevance, whereby a gap in the recent literature exists. The structured manual and procedure, as well as the reasonable cohort, are strengths of this study. On the one hand, the power of the sample appears unclear; especially regarding the potential relevance of the risk classification and patient screening for EP infections, no robust conclusions can be drawn. No sample size calculation was performed, and the sample was limited, as only patients from the previous study could be included. Considering the low prevalence of infectious complications after EP surgery, the number of patients in the current study seems too low to draw clinical consequences from the current study. Thus, the results must be seen as preliminary. Additionally, the information on dental visits and respective therapy was only provided by the patients. Therefore, only self-reported information about the patients was available, making unclear what kind of dental measures were exactly performed and whether the potential oral foci were really eliminated. To verify the results, a clinical dental examination would have been required in follow-up, but this was not possible for organizational reasons. The high number of dropouts is a potential bias of the sample, whereby especially patients, which did not visit the dentists, might have been not included. Accordingly, the current findings need to be confirmed by further large-scaled, prospective clinical studies. 

## 5. Conclusions

After dental referral, most patients prior to EP visit their general dentist, especially if they have a potential oral focus. The effect of dental screening and risk classification on infectious complications of the EP remains unclear, whereby further large-scaled, prospective clinical studies are needed. 

## Figures and Tables

**Table 1:** Patients’ characteristics before EP implantation.

		Overall	LR Group	MR Group	HR Group	*p*-Value
number of patients (*n* [%])		96 (100)	19 (20)	41 (43)	36 (37)	--
age in years (mv ± sd)		67.6 ± 10.1	72.4 ± 8.9	66.8 ± 10.9	65.9 ± 9.0	*p* = 0.06
gender (male, *n*)		51	8	23	20	*p* = 0.56
joint prothesis (*n*)	knee	33	5	16	12	*p* = 0.62
	hip	63	14	25	24	
Time point after joint surgery in month (median [IQR])		15 (5.5)	15 (5)	16 (3)	15 (2.5)	*p* = 0.20

EP: endoprothesis; LR: low risk; MR: moderate risk; HR: high risk; *n*: number; IQR: inter quartil range.

**Table 2:** Number of complications and needs-based interventions after joint surgery (*n*).

		Overall (*n* = 96)	LR Group (*n* = 19)	MR Group (*n* = 41)	HR Group (*n* = 36)	*p*-Value
occurred complication	overall	14	4	4	6	*p* = 0.51
	infected	4	2	1	1	*p* = 0.30
	WHD	0	0	0	0	-
	loosening	1	1	0	0	*p* = 0.13
	other	9	2	3	4	*p* = 0.84
Time point of complication	<4 weeks	6	2	2	2	*p* = 0.69
	>4 weeks	2	1	0	1	*p* = 0.39
intervention	antibiosis	4	2	1	1	*p* = 0.30
	surgical (re-)treatment	4	1	2	1	*p* = 0.87
	one-stage change	1	0	1	0	*p* = 0.51
	two-stage change	0	0	0	0	-
	no information	5	-	-	-	-

LR: low risk, MR: moderate risk, HR: high risk, *n*: number, WHD: wound healing disorder.

## Data Availability

The datasets used and/or analyzed during the current study are available from the corresponding author upon reasonable request. The data are not publicly available, because of the pseudonymization and data protection guidelines according to the ethics approval.
